# Genome-Wide Gene Expression Profiling Revealed a Critical Role for GATA3 in the Maintenance of the Th2 Cell Identity

**DOI:** 10.1371/journal.pone.0066468

**Published:** 2013-06-18

**Authors:** Tetsuya Sasaki, Atsushi Onodera, Hiroyuki Hosokawa, Yukiko Watanabe, Shu Horiuchi, Junji Yamashita, Hitoshi Tanaka, Yasumasa Ogawa, Yutaka Suzuki, Toshinori Nakayama

**Affiliations:** 1 Department of Immunology, Graduate School of Medicine, Chiba University, Chiba City, Japan; 2 Pharmaceutical Research Laboratory, Research Division, Nihon Pharmaceutical Co Ltd, Narita City, Japan; 3 Laboratory of Functional Genomics, Department of Medical Genome Sciences, Graduate School of Frontier Sciences, University of Tokyo, Kashiwa City, Japan; 4 JST, CREST, Chiba City, Japan; Juntendo University School of Medicine, Japan

## Abstract

Functionally polarized CD4+ T helper (Th) cells such as Th1, Th2 and Th17 cells are central to the regulation of acquired immunity. However, the molecular mechanisms governing the maintenance of the polarized functions of Th cells remain unclear. GATA3, a master regulator of Th2 cell differentiation, initiates the expressions of Th2 cytokine genes and other Th2-specific genes. GATA3 also plays important roles in maintaining Th2 cell function and in continuous chromatin remodeling of Th2 cytokine gene loci. However, it is unclear whether continuous expression of GATA3 is required to maintain the expression of various other Th2-specific genes. In this report, genome-wide DNA gene expression profiling revealed that GATA3 expression is critical for the expression of a certain set of Th2-specific genes. We demonstrated that GATA3 dependency is reduced for some Th2-specific genes in fully developed Th2 cells compared to that observed in effector Th2 cells, whereas it is unchanged for other genes. Moreover, effects of a loss of GATA3 expression in Th2 cells on the expression of cytokine and cytokine receptor genes were examined in detail. A critical role of GATA3 in the regulation of Th2-specific gene expression is confirmed in *in vivo* generated antigen-specific memory Th2 cells. Therefore, GATA3 is required for the continuous expression of the majority of Th2-specific genes involved in maintaining the Th2 cell identity.

## Introduction

Naïve CD4 T cells have the capacity to differentiate into several alternative cell types, the best characterized of which are Th1, Th2 and Th17 cells [1]. Th1 cells are crucial for obtaining protection against viruses and intracellular pathogens and Th2 cells are required for the removal of extracellular parasites. Th1 cells are involved in the pathogenesis of tissue-specific autoimmune diseases, while Th2 cells are responsible for allergic diseases such as asthma. Th17 cells function in the immune response to extracellular bacteria and participate in the development of inflammatory bowel diseases. Several master transcription factors that regulate Th1/Th2/Th17 cell differentiation have been identified. STAT1 and STAT4 induce the expression of T-bet and act as key regulators of Th1 cell fate determination [2]. The generation of Th2 cells requires IL-4, which leads to STAT6 phosphorylation [3] and the upregulation of GATA3, the key regulator of Th2 development [4], [5]. Th17 development is thought to be dependent on the lineage-specific transcription factor retinoic acid-related orphan receptor (ROR) γt [6], [7].

During the differentiation of these Th subsets, the expression of a certain set of genes, including a specific master transcription factor, is upregulated, which in turn leads to the expression of subset-specific cytokine genes. Furthermore, a more fundamental regulator of gene expression during Th cell differentiation, so-called chromatin remodeling, establishes the stable expression of subset-specific cytokine genes [8]. Once these cell subsets have been established, they lose the potential to differentiate into other subsets. However, it has been reported that, under certain conditions, some Th cell subsets, particularly Th17 cells, preserve a substantial capacity for re-differentiation called plasticity [9].

In peripheral CD4 T cells, the activation of STAT6 induces high-level GATA3 mRNA expression [10]. Additionally, we and others have recently reported that STAT6 binds to specific regions of the GATA3 gene to regulate its transcription [11], [12]. Changes in active histone modifications such as H3-K9/14 acetylation and H3-K4 methylation at Th2 cytokine gene loci occur during Th2 cell differentiation [13], [14], [15], and are induced primarily by GATA3 in both CD4 and CD8 T cells [16]. Several reports, including ours, have shown that GATA3 plays important roles in continuous chromatin remodeling of the specific Th2 cytokine gene locus and in the maintenance of the ability to produce large amount of the Th2 cytokines [17], [18], [19], [20]. A high-level expression of GATA3 is strictly maintained in fully developed Th2 cells in a Menin/Trithorax-dependent but IL-4/STAT6-independent manner, indicating that Th2 cells possess relatively low plasticity [12]. Recently, genome-wide studies using chromatin immunoprecipitation (ChIP) assays coupled with massive parallel sequencing analyses (ChIP-Seq) have identified many of the GATA3-bound regions in several CD4 T cell subsets [21] [22]. However, it is still unclear whether the continuous expression of GATA3 is required for the maintenance of Th2-specific gene expression to preserve Th2 cell identity.

In order to study the role of GATA3 in the maintenance of Th2 cell identity and function, we identified GATA3-regulated genes in both effector Th2 cells and fully developed Th2 cells using microarray analyses. Of the GATA3-regulated gene candidates, we selected 65 after taking into account information about gene product function and our previously deposited ChIP-Seq datasets. Gene expression profiling of these 65 genes not only showed the importance of GATA3 in fully developed Th2 cells, but also provided interesting information about the behavior of gene expression. Finally, we assessed the effects of GATA3 knockdown on memory Th2 cells. We observed that GATA3 regulates the expression of the majority of Th2-specific genes, not only in fully developed Th2 cells, but also in antigen-specific memory Th2 cells. Therefore, these approaches revealed multiple manners in which GATA3 is involved in the maintenance of the Th2 cell identity.

## Materials and Methods

### Mice

C57BL/6 mice were purchased from CLEA (Tokyo, Japan). All mice used in this study were maintained under specific pathogen-free conditions and ranged from six to eight weeks in age. All animal care was performed in accordance with the guidelines of Chiba University. Experimental animals were sacrificed by cervical dislocation. We made every efforts to relieve the pain of experimental animals. The protocol was approved by the Committee on the Ethics of Animal Experiments of Chiba University (Permit Number: 24-4).

### Reagents

Recombinant murine IL-12 was purchased from BD Pharmingen and recombinant murine IL-4 was purchased from TOYOBO (Osaka, Japan).

### Antibodies

The antibodies used for the ChIP assay included anti-GATA3 mAbs (Santa Cruz Biotechnology) and anti-acetylhistone H3-K9 (Upstate; 06–599).

### Generation of Effector Th1 and Th2 Cells

Effector Th1/Th2 cells were generated as previously described [23]. Splenic CD4 T cells were prepared using a magnetic cell sorter (Auto-MACS; Miltenyi Biotec) yielding a purity of >98%. Where indicated, cells from C57BL/6 mice were stimulated with immobilized anti-TCR mAbs (H57–597; 5µg/ml) and anti-CD28 mAbs under Th1 or Th2 culture conditions for five days *in vitro*. The Th1 conditions were as follows: 25 U/ml of IL-2, 10 U/ml of IL-12 and anti–IL-4 mAbs. The Th2 conditions were as follows: 25 U/ml of IL-2 and 100 U/ml of IL-4. The cells were used as either Th1 or Th2 cells, respectively.

### Establishment of Fully Developed Th2 Cells

Splenic CD4 T cells were stimulated under Th2 culture conditions for five days *in vitro*. The Th2 cells were further cultured *in vitro* for another two days in the absence of any exogenous cytokines. The cultured CD4 T cells were then restimulated with immobilized anti-TCR mAbs with IL-2 and anti-IL-4 mAbs for 5 days. We defined these cells as fully developed Th2 cells [12]. This cycle was repeated more than three times. (Th2-2^nd^ ∼ Th2-4^th^).

### Generation of Memory Th2 Cells

Memory Th2 cells were generated as previously described [23]. In brief, Splenic CD4 T cells from DO11.10 OVA-specific TCR Tg mice were stimulated with OVA peptides (Loh15, 1µM) plus APC under Th2-culture conditions for six days *in vitro*. The effector Th2 cells (3 × 10^7^) were transferred intravenously into BALB/c recipient mice. Five weeks after cell transfer, splenic KJ1+ cells were purified using a cell sorter (BD FACSAria) and then used as memory Th2 cells.

### Microarray Data Collection and Analysis

Total RNA from cells treated with GATA3 siRNA or control siRNA was extracted with the TRIzol reagent (Invitrogen) according to the manufacturer’s instructions. Approximately 5 µg of RNA was labeled and hybridized to GeneChip Mouse Genome 430 2.0 arrays (Affymetrix) according to the manufacturer’s protocol. The expression values were determined with the Affymetrix GeneChip Command Console Software (AGCC) and Console Software (Expression Console).

### Quantitative PCR Analysis

For the PCR studies, total RNA was isolated using the TRIzol reagent (Invitrogen) and cDNA was synthesized using oligo(dT) primers and Superscript II RT (Invitrogen). Quantitative RT-PCR was performed as previously described using the ABI PRISM 7500 Sequence Detection System [23]. The primers and TaqMan probes used for the amplification and detection of the indicated genes and the *Hprt* gene were purchased from Applied Biosystems and Roche, respectively. The expression levels of the target genes were normalized to those of the *Hprt* signals. For quantitative RT-PCR (qRT-PCR), cells restimulated by anti-TCR mAbs (H57–597; 3µg/ml) for four hours were used. Statistical analysis was performed using following three criteria.

- 10^−0.5^ or 10^0.5^-fold change by GATA3 knockdown
- p<0.05 (Student’s t-test) with real time PCR performed with duplicate samples
- Representative results were obtained from two independent experiments.

### Knockdown Assay

To knock down the *Gata3* genes, we used the Mouse T cell Nucleofector Kit (Amaxa) according to the manufacturer’s protocol. The siRNAs used for the knockdown of GATA3 (s66482) and the negative control (AM4635) were purchased from Applied Biosystems.

### ChIP-Seq and Illumina Sequencing

For the ChIP-Seq analyses, IP and input samples were prepared using a ChIPSeq Sample Prep kit (Illumina). Adaptor-ligated DNA fragments were size fractionated using 12% acrylamide gels, and the 170- to 250-bp fractions were recovered. The DNA obtained was amplified with 18 cycles of PCR. One nanogram of DNA was used for the sequencing reaction in the Illumina GAIIx according to the manufacturer’s instructions. A total of 170,000–250,000 clusters were generated per tile, and 36 cycles of the sequencing reactions were performed. The short-read sequences were aligned to the murine genome sequences (mm9 from the University of California, Santa Cruz Genome Browser; http://genome.ucsc.edu/) using the Eland program. Sequences allowing no more than two mismatches per sequence were used for the analysis. To enumerate the GATA3-bound genes, at least one peak (with a five-fold increase in signal intensity compared with input DNA) detected on the gene locus was selected.

### Accession Number

The ChIP-Seq data sets of the histone modifications and the microarray data for the Th1 and Th2 cells are available in the Gene Expression Omnibus (GEO) database (http://www.ncbi.nlm.nih.gov/geo) under accession number GSE28292. The microarray data for the GATA3 knockdown cells is available in the GEO database under accession number GSE46185.

## Results

### Selection of Th2-specific Inducible Genes

To investigate the role of GATA3 in the induction and maintenance of Th2 cell identity, we used effector Th2 cells and fully developed Th2 cells, respectively. Hereafter, we refer to the effector Th2 cells generated with *in vitro* cultivation for five days as Th2 or Th2-1^st^. The Th2 cells generated *in vitro* with various cycles of stimulation with antigens under conditions in which IL-4 was blockaded by antibodies (Th2-2^nd^ ∼ Th2-4^th^; see Materials and Methods) are referred to as fully developed Th2 cells. We previously reported that Th2 cell identity was maintained in an IL-4-independent manner [12].

**Figure 1:**
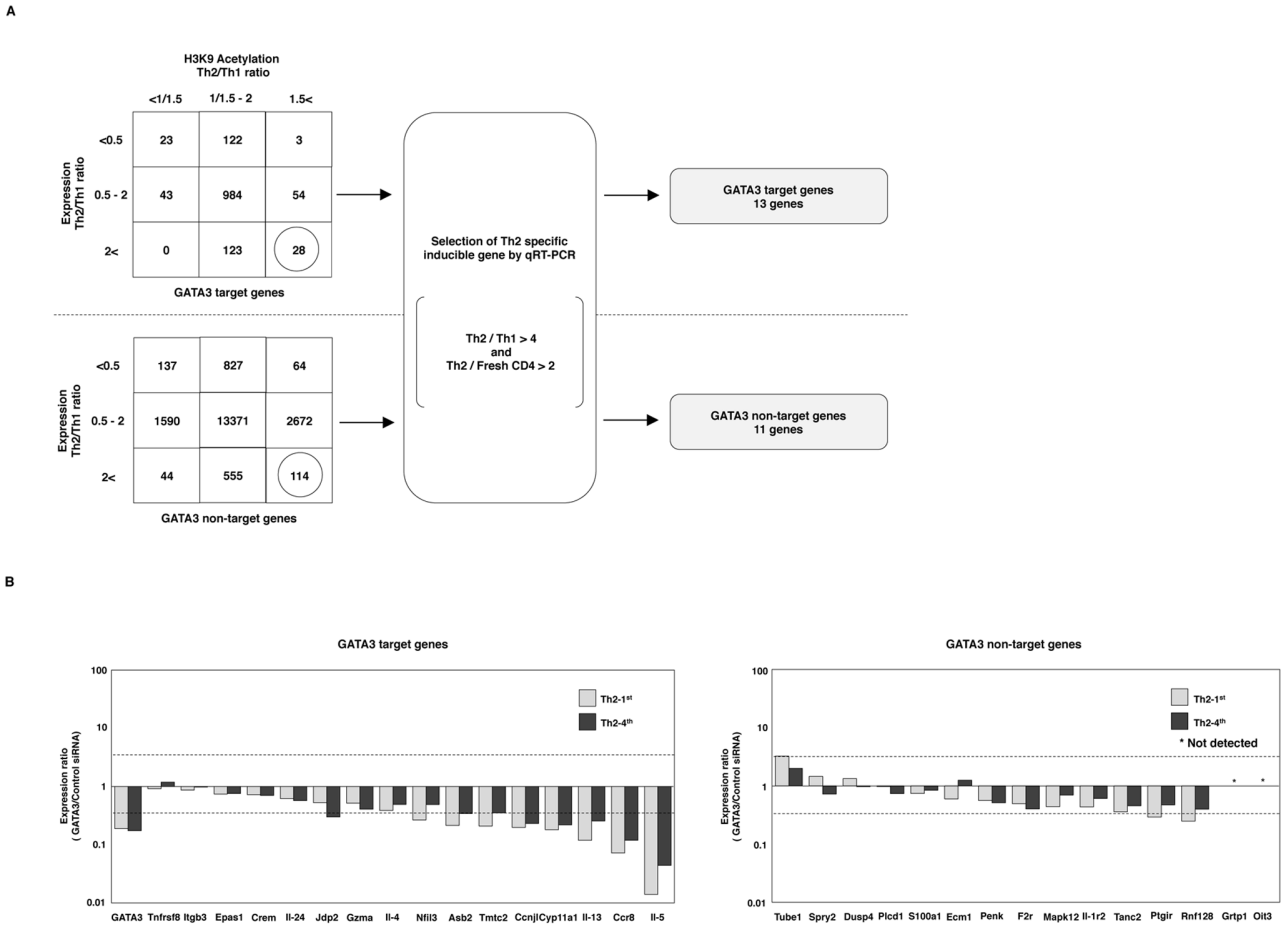
The effects of GATA3 knockdown on the expression of Th2-specific genes. **(A),** GATA3 target and non-target genes were classified into nine groups according to their H3K9Ac and expression levels in Th2 cells. The H3K9Ac levels were determined according to the total tag count of H3K9Ac across the gene body of each GATA3-target and GATA3-non-target RefSeq gene in Th2 versus Th1 cells. The right column indicates the number of genes that showed more than 1.5-fold Th2/Th1 ratios for the H3K9Ac level. The center indicates 1/1.5 to 1.5-fold Th2/Th1 ratios and the left column indicates genes with ratios of less than 1/1.5-fold. The transcription levels were determined using a microarray analysis that compared Th2 to Th1 cells. The lower row indicates the number of genes that showed more than two-fold Th2/Th1 ratios in expression. The middle row indicates 0.5 to two-fold Th2/Th1 ratios and the upper row indicates less than 0.5-fold ratios. From this analysis, 28 potential Th2-specific GATA3 target genes and 114 potential Th2-specific GATA3 non-target genes were identified (lower right squares in each table). The 28 potential Th2-specific GATA3 target genes and the 114 potential Th2-specific GATA3 non-target genes were validated with qRT-PCR. Next, Th2-high expression (Th2/Th1>4) and inducible (Th2/Fresh-CD4>2) genes were selected. Finally, 11 Th2-specific GATA3 non-target genes and 13 GATA3 target genes were identified. **(B),** The effects of GATA3 knockdown on Th2-1st and fully developed Th2 cells (Th2-4^th^) were evaluated. The mRNA expression of the Th2-specific genes in these cells was determined with qRT-PCR. The relative expression (GATA3/Control siRNA) levels are shown. The lower dashed line indicates 10^−1/2^ and the upper dashed line indicates 10^1/2^.

First, we assessed the effects of knocking down GATA3 on the expression of various Th2-specific genes. To identify the Th2-specific genes whose transcription levels are upregulated during Th2 cell differentiation and are preferentially higher in Th2 cells than in Th1 cells, we adopted a two-step selection process (Fig. 1A). During the first step, we classified GATA3 target and non-target genes into nine groups according to their H3K9Ac and mRNA expression levels in Th2 cells. The H3K9Ac levels were determined according to the total tag count of H3K9Ac across the gene body of each GATA3 target and GATA3 non-target RefSeq gene in Th2 versus Th1 cells. The transcription levels in Th2 and Th1 cells were determined and compared using a microarray analysis. To summarize, the genes divided into the lower right square were upregulated transcriptionally and epigenetically in Th2 cells. From this analysis, we identified 28 potential Th2-specific GATA3 target genes and 114 potential Th2-specific GATA3 non-target genes. During the second step, the 28 potential Th2-specific GATA3 target genes and 114 potential Th2-specific GATA3 non-target genes were validated with qRT-PCR and the Th2 high expression (Th2/Th1>4) and inducible (Th2/Fresh-CD4>2) genes were selected (Fig. S1). In this step, we identified 13 Th2-specific GATA3 target genes (Ccnjl, Ccr8, Cyp11a1, Epas1, GATA3, Gzma, Il-13, Il-24, Il-4, Il-5, Itgb3, Tmtc2 and Tnfrsf8) and 11 GATA3 non-target genes (Ecm1, Grtp1, Il-1r2, Mapk12, Oit3, Penk, Plcd1, Rnf128, S100a1, Spry2 and Tube1) (Fig. 1A). We have previously reported the identification of 26 Th2-specific genes [22]. Unifying both the 24 genes identified in the present study and the previously reported 26 genes, we defined 32 (GATA3, Tnfrsf8, Itgb3, Epas1, Crem, Il-24, Jdp2, Gzma, Il-4, Nfil3, Asb2, Tmtc2, Ccnjl, Cyp11a1, Il-13, Ccr8, Il-5, Tube1, Spry2, Dusp4, Plcd1, S100a1, Ecm1, Penk, F2r, Mapk12, Il-1r2, Tanc2, Ptgir, Rnf128, Grtp1 and Oit3) genes as Th2-specific inducible genes.

### Effects of GATA3 Knockdown on Th2-specific Gene Expression

The GATA3 dependency of these 32 selected Th2-specific genes was examined using GATA3 siRNA in Th2-1^st^ and Th2-4^th^ cells. The expressions of almost all GATA3 target genes, except Tnfrsf8, were decreased by GATA3 knockdown in both the Th2-1^st^ and Th2-4^th^ cells (Fig. 1B, left). GATA3 protein levels in GATA3 siRNA-transfected cells were significantly decreased in Th2-1^st^ and Th2-4^th^ cells compared with control cells (Fig. S2). Hereafter, a substantial increase is defined as a more than 10^1/2^-fold (3.16-fold) increase in expression induced by GATA3 knockdown. Likewise, a substantial decrease is defined as a less than 10^−1/2^-fold (0.316-fold) decrease in expression induced by GATA3 knockdown. For instance, the expression levels of Nfil3, Asb2, Tmtc2, Ccnjl, Cyp11a1, Il-13, Ccr8 and Il-5 decreased to less than 31.6% (Fig. 1B, the lower dashed line indicates 10^−1/2^). Therefore, we defined these genes as substantially decreased genes. Among the GATA3 target genes, eight (Nfil3, Asb2, Tmtc2, Ccnjl, Cyp11a1, Il-13, Ccr8 and Il-5) of 16 (50.0%) were found to be substantially decreased in the Th2-1^st^ cells, while six (Jdp2, Ccnjl, Cyp11a1, Il-13, Ccr8 and Il-5) of 16 (37.5%) were found to be substantially decreased in Th2-4^th^ cells. In contrast, the GATA3 non-target genes responded in different ways. Some were upregulated, while others were downregulated or not changed, by GATA3 knockdown (Fig. 1B, right). The Tube1 expression level was substantially increased (more than 3.16-fold), whereas the Ptgir and Rnf128 expression levels were substantially decreased, in the Th2-1^st^ cells. We could not detect Oit3 or Grtp1 mRNA using qRT-PCR. In conclusion, in Th2-1^st^ cells, one (Tube1) of 13 (7.7%) genes was substantially increased, whereas two (Ptgir and Rnf128) of 13 (15.4%) were substantially decreased. No genes were substantially changed in the Th2-4^th^ cells. These results indicate that GATA3 plays a critical role in the expression of the Th2-specific genes in both Th2-1^st^ and Th2-4^th^ cells, and that GATA3 target genes appeared to be more sensitive to the loss of GATA3 than the GATA3 non-target genes.

### GATA3-regulated Genes Identified by Microarray Analysis of GATA3 siRNA-treated Th2 Cells

**Figure 2:**
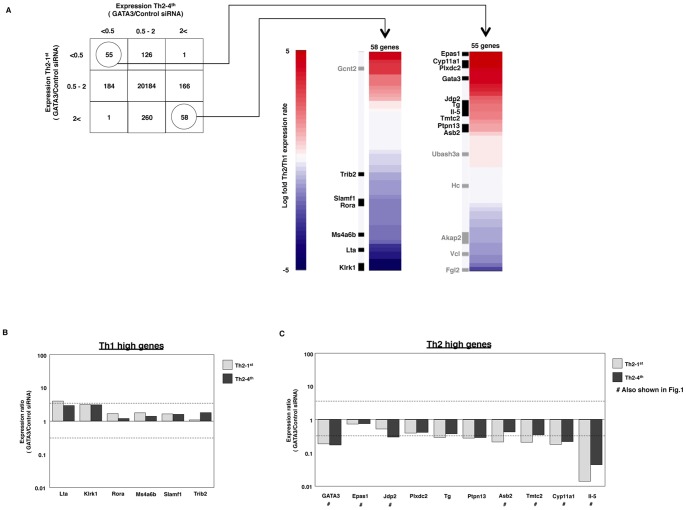
The microarray analysis revealed the potential GATA3-regulated genes in Th2 cells. (**A**), Effector Th2 cells (1^st^) and fully developed Th2 cells (4^th^) were treated with GATA3 siRNA and their gene expression profiles were studied and compared to cells treated with control siRNA using a microarray analysis. In the upper panel, the lower row indicates the number of genes that showed more than two-fold GATA3/Control siRNA ratios in the Th2-1^st^ cells. The middle row indicates 0.5 to two-fold GATA3/Control siRNA ratios and the upper row indicates less than 0.5-fold ratios. The right column indicates the number of genes that showed more than two-fold GATA3/Control siRNA ratios in Th2-4^th^ cells. The center indicates a 0.5 to two-fold GATA3/Control siRNA ratios and the left column indicates ratios of less than 0.5-fold. A total of 55 genes were identified as being positively regulated by GATA3 in both Th2 and Th2-4^th^ cells in the microarray analysis. Another 58 genes were determined to be negatively regulated by GATA3 in both Th2 and Th2-4^th^ cells. The right heat map shows the classification of the 55 common positively regulated genes and the 58 common negatively regulated genes. The black and gray bars indicate the genes in which GATA3 binding was detected in the ChIP-seq analysis. Among these 55 positively regulated genes, 10 GATA3 target genes were classified as Th2-high genes and seven GATA3 target genes were classified as Th1-high genes. (**B**), The effects of GATA3 knockdown on the genes negatively regulated by GATA3 in Th2-1^st^ and Th2-4^th^ cells were evaluated with qRT-PCR. The relative expression (GATA3/Control siRNA) levels are shown. The lower dashed line indicates 10^-1/2^ and the upper dashed line indicates 10^1/2^. (**C**), The effects of GATA3 knockdown on the genes positively regulated by GATA3 in Th2-1^st^ and Th2-4^th^ cells were evaluated with qRT-PCR. The relative expression (GATA3/Control siRNA) levels are shown. A number sign (#) with the gene name indicates data that are also shown in Figure 1.

We next performed a microarray analysis using control and GATA3 siRNA-treated Th2-1^st^ and Th2-4^th^ cells to identify the GATA3-regulated genes in genome wide. Consequently, in the Th2-1^st^ cells, 182 (55+126+1) genes were found to be downregulated and 319 (1+260+58) genes were found to be upregulated by GATA3 knockdown (see Fig. 2A). We also identified 240 (55+184+1) downregulated genes and 225 (1+166+58) upregulated genes in the Th2-4^th^ cells. We found 55 genes whose expression levels were positively regulated by GATA3 in both Th2-1^st^ and Th2-4^th^ cells in the microarray analysis. There were also 58 genes whose expression levels were negatively regulated by GATA3 in both Th2-1^st^ and Th2-4^th^ cells. Next, we classified the 55 positively regulated genes and the 58 negatively regulated genes based on the results of GATA3 binding to the gene loci and the Th2/Th1 expression ratio. We found that 10 (Epas1, Cyp11a1, Plxdc2, GATA3, Jdp2, Tg, Il-5, Tmtc2, Ptpn13 and Asb2) of the 55 positively regulated genes were GATA3 targets, and seven (Trib2, Slamf1, Rora, Ms4a6b, Lta and Klrk1) of the 58 negatively regulated genes were classified as GATA3 targets (Fig. 2A, right panels). These 17 genes encode 16 unique genes, because the Klrk1 gene has two RefSeq ID numbers.

### Effects of GATA3 Knocking Down on GATA3 Target Genes

To confirm the negative (repressive) or positive (enhancing) effects of GATA3, we knocked down GATA3 in Th1 and Th2 cells using siRNA and the mRNA expression levels of the above identified 16 GATA3 target genes were determined with qRT-PCR (Fig. S3). We observed that all six negatively regulated genes (Th1 high genes) were derepressed in Th2 cells by GATA3 knockdown (Fig. 2B). In particular, the Lta gene expression was substantially increased in Th2-1^st^ cells by GATA3 knockdown. Decreased expression was observed in 10 positively regulated genes (Th2 high genes) in Th2 cells (Fig. 2C). Among the Th2 high genes, six (Tg, Ptpn13, Asb2, Tmtc2, Cyp11a1 and Il-5) of 9 (66.7%) genes were found to be substantially decreased in Th2-1^st^ cells.

We also assessed whether GATA3 had a negative or positive effect on the expression of these genes in fully developed Th2-4^th^ cells. The de-repression of the gene expression was detected in six negatively regulated genes (Th1 high genes) (Fig. 2B). No genes were found to be substantially increased in the Th2-4^th^ cells. GATA3 knockdown decreased the expression levels of nine positively regulated genes in fully developed Th2-4^th^ cells (Fig. 2C). Four (Jdp2, Ptpn13, Cyp11a1 and Il-5) of nine (44.4%) genes were found to be substantially decreased in the Th2-4^th^ cells. These results indicate that GATA3 has a repressive effect on Th1-high genes, whereas it induces the expression of Th2-high genes in both Th2 and Th2-4^th^ cells.

### Cytokine and Cytokine Receptor Genes Regulated by GATA3 in Th2 Cells

**Figure 3:**
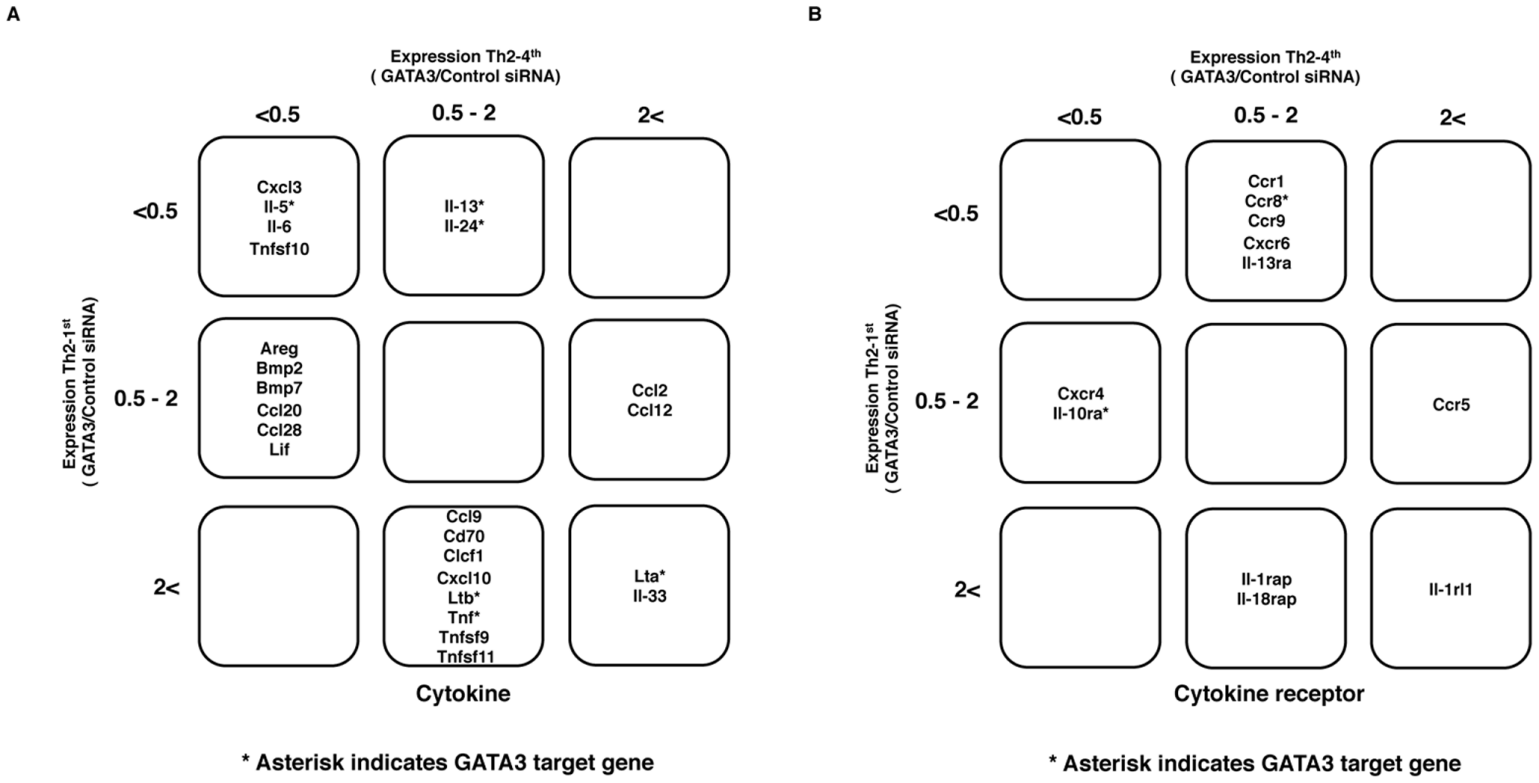
The results of the microarray analysis revealed the GATA3-regulated cytokine and cytokine receptor genes in Th2 cells. (**A**), The names of the selected 24 cytokine genes. The decreased expression of four genes (Cxcl3, Il-5, Il-6 and Tnfsf10) was observed in both Th2-1^st^ and Th2-4^th^ cells. GATA3 siRNA reduced the expression of two genes (Il-13 and Il-24) in Th2-1^st^ cells and six genes (Areg, Bmp2, Bmp7, Ccl20, Ccl28 and Lif) in Th2-1^st^ cells. The increased expression of two genes (Lta and Il-33) was observed in both Th2 and Th2-4^th^ cells. GATA3 siRNA increased the expression of eight genes (Ccl9, Cd70, Clcf1, Cxcl10, Ltb, Tnf, Tnfsf9 and Tnfsf11) in Th2-1^st^ cells and two genes (Ccl2 and Ccl12) in Th2-4^th^ cells. (**B**), The names of 11 selected cytokine receptor genes. GATA3 siRNA reduced the expression of five genes (Ccr1, Ccr8, Ccr9, Cxcr6 and Il-13ra) in Th2-1^st^ cells, and two genes (Cxcr4 and Il-10ra) in Th2-4^th^ cells. The increased expression of one gene (Il-1rl1) was observed in both Th2-1^st^ and Th2-4^th^ cells. GATA3 siRNA increased the expression of two genes (Il-1rap and Il-18rap) in Th2-1^st^ cells and one gene (Ccr5) in Th2-4^th^ cells.

CD4 T cells express various kinds of cytokines and cytokine receptors to regulate Th cell differentiation and polarized Th cell-mediated immune responses. We next investigated the negative or positive effects of GATA3 on the expression of cytokine and cytokine receptor genes in detail. We looked back at the microarray data shown in the left panel of Fig. 2A and picked up the cytokine and cytokine receptor genes among the 365 decreased genes (55+126+184) and 484 increased genes (58+260+166) (Fig. 3). A gene ontology (GO) functional annotation for these decreased and increased genes was performed using the DAVID analysis tool (http://david.abcc.ncifcrf.gov/home.jsp). Twenty-four cytokine genes and 11 cytokine receptor genes were identified. Consequently, we found 12 cytokine genes whose expressions were found to be positively regulated by GATA3 (Cxcl3, Il-5, Il-6, Tnfsf10, Il-13, Il-24, Areg, Bmp2, Bmp7, Ccl20, Ccl28 and Lif). Our data also identified another 12 cytokine genes whose expressions were found to be negatively regulated by GATA3 (Lta, Il-33, Ccl9, Cd70, Clcf1, Cxcl10, Ltb, Tnf, Tnfsf9, Tnfsf11, Ccl2, and Ccl12) (Fig. 3A). Our analysis demonstrated that seven cytokine receptor genes are positively regulated by GATA3 (Ccr1, Ccr8, Ccr9, Cxcr6, Il-13ra, Cxcr4 and Il-10ra) and four cytokine receptor genes are negatively regulated by GATA3 (Il-1rl1, Il-1rap, Il-18rap and Ccr5) (Fig. 3B).

### Effects of GATA3 Knockdown on the Expression Levels of Cytokine and Cytokine Receptor Genes

**Figure 4:**
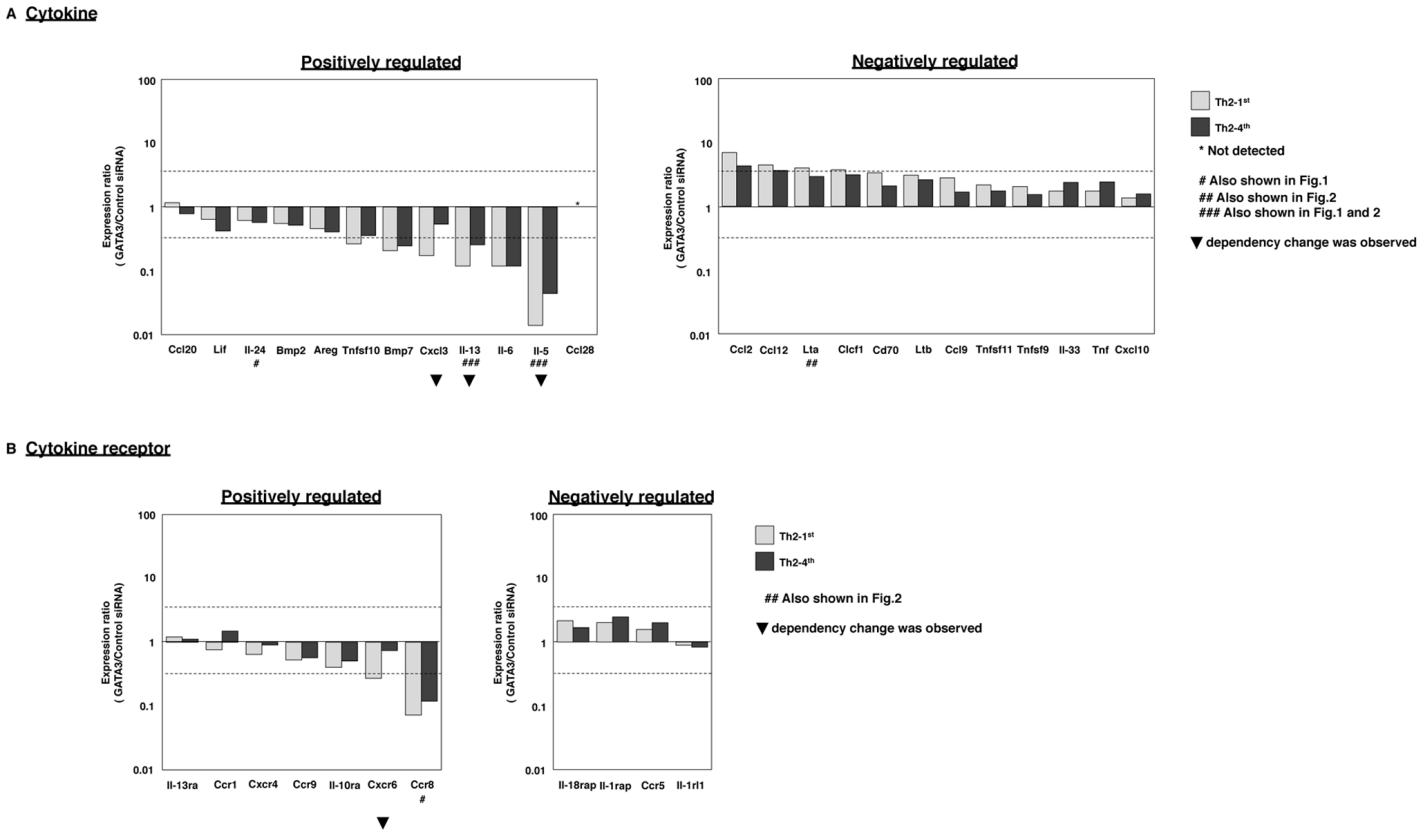
The effects of GATA3 knockdown on the expression of cytokine and cytokine receptor genes. The effects of GATA3 knockdown on cytokine (A) and cytokine receptor (B) gene expression in Th2-1^st^ and Th2-4^th^ cells were evaluated with qRT-PCR. The relative expression (GATA3/Control siRNA) levels of the cytokine and cytokine receptor genes are shown. A number sign (#) with the gene name indicates data that are also shown in previous Figures. The filled triangles indicate the genes that showed GATA3 dependency changes (more than two-fold) in Th2-4^th^ cells compared with that observed in Th2-1^st^ cells. The lower dashed line indicates 10^-1/2^ and the upper dashed line indicates 10^1/2^.

The GATA3 dependency of the 24 cytokine and 11 cytokine receptor genes was examined using GATA3 siRNA in Th2-1^st^ and Th2-4^th^ cells (Fig. S4). The expression ratios (GATA3/Control siRNA) of the cytokine and cytokine receptor genes were determined using qRT-PCR. As shown in the left panel of Fig. 4A, most of the cytokine genes, classified as being positively regulated genes showed decreased expression levels following GATA3 knockdown. GATA3 knockdown had a weak effect on the Ccl20 expression, and we were unable to detect Ccl28 mRNA in TH2 cells with qRT-PCR. Among positively regulated cytokine genes, six (Tnfsf10, Bmp7, Cxcl3, Il-13, Il-6 and Il-5) of 12 (50.0%) were found to be substantially decreased in Th2-1^st^ cells and four (Bmp7, Il-13, Il-6 and Il-5) of 12 (33.3%) were found to be substantially decreased in Th2-4^th^ cells. We also found that GATA3 dependency was reduced by more than two-fold in the Cxcl3, Il-5 and Il-13 genes (filled triangles). In contrast, the expression levels of the 12 cytokine genes classified as negatively regulated genes were increased by GATA3 siRNA (Fig. 4A, right). Among the negatively regulated genes, five (Ccl2, Ccl12, Lta, Clcf1 and Cd70) of 12 (41.7%) cytokines were found to be substantially increased in Th2-1^st^ cells and two (Ccl2 and Ccl12) of 12 (16.7%) were found to be substantially increased in Th2-4^th^ cells.

We next examined the cytokine receptor genes classified as GATA3 positively regulated genes (Fig. 4B, left). The Ccr8 expression level especially decreased in both Th2-1^st^ and Th2-4^th^ cells. Therefore, a high level of GATA3 dependency was sustained in the Ccr8 gene. Among positively regulated cytokine receptors, two (Cxcr6 and Ccr8) of seven (28.6%) receptors were found to be substantially decreased in Th2-1^st^ cells and one (Ccr8) of seven (14.3%) receptors was found to be substantially decreased in Th2-4^th^ cells. In contrast, the expression levels of the three cytokine receptor genes classified as negatively regulated genes were increased approximately two-fold by GATA3 siRNA (Fig. 4B, right). The Il-1rl1 gene expression was slightly decreased by GATA3 knockdown. Among negatively regulated cytokine receptors, no genes substantially increased in either Th2-1^st^ or Th2-4^th^ cells.

A decrease in GATA3 dependency of more than two-fold was observed in the Cxcr6 gene (filled triangles). Taken together, these results indicate that GATA3 dependency was comparable between Th2 and Th2-4^th^ cells, although for four genes (Cxcl3, Il-13, Il-5 and Cxcr6), GATA3 dependency was decreased by more than two-fold (Fig. S5).

### Comparison of GATA3 Dependency in Th2-1^st^, Th2-2^nd^, Th2-3^rd^ and Th2-4^th^ Cells

**Figure 5:**
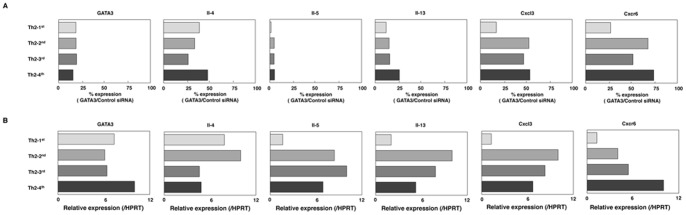
Decreases in GATA3 dependency correlate with increases in the gene expression. (**A**), The effects of GATA3 knockdown on effector Th2 cells (1st) and fully developed Th2 cells (2nd, 3rd and 4th) were determined with qRT-PCR. The % expression (GATA3/Control siRNA) was found to be slightly increased in Il-5, Il-13, Cxcl3 and Cxcr6 in accordance with the culture cycle number. (**B**), The gene expression levels of Th2 cells (1st) and fully developed Th2 cells (2nd, 3rd and 4th) were determined with qRT-PCR. The gene expression levels of Il-5, Il-13, Cxcl3 and Cxcr6 were increased in fully developed Th2 cells compared with those observed in effector Th2 cells.

We next compared quantitatively the GATA3 dependency of GATA3, Il-4 and the above detected 4 genes (Cxcl3, Il-13, Il-5 and Cxcr6) in effector Th2 cells (Th2-1^st^) with that observed in fully developed Th2 cells (Th2-2^nd^ - 4^th^) (Fig. 5A). In the Il-5, Il-13, Cxcl3 and Cxcr6 genes, the effect of knocking down GATA3 on the gene expression (GATA3 dependency) decreased in accordance with the culture cycle number. In the Il-4 genes, no obvious GATA3 dependency changes were observed in relation to the culture cycle number. We next determined the relative gene expression levels of these six genes using qRT-PCR (Fig. 5B). For GATA3, the expression levels were comparable between effector Th2 cells and fully developed Th2 cells. On the other hand, for the Il-5, Il-13, Cxcl3 and Cxcr6 genes, the expression levels increased by more than two-fold in fully developed Th2 cells compared with those observed in effector Th2 cells. The degree of GATA3 binding at all target genes was almost comparable regardless of the development stage of Th2 cells (Th2-1^st^ to Th2-4^th^) (Fig. S6). These results indicate that GATA3 dependency is negatively correlated with the mRNA expression levels of these four genes.

### Effects of GATA3 Knockdown on the Gene Expression of in vivo Generated Memory Th2 Cells

**Figure 6:**
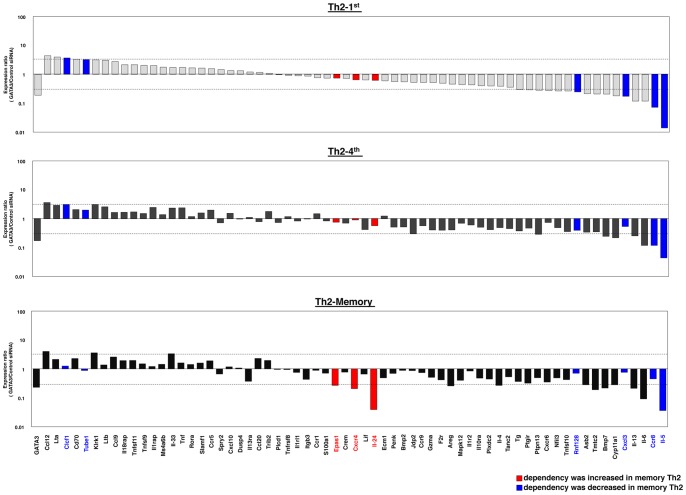
The effects of GATA3 knockdown on the gene expression of Th2-1^st^, Th2-4^th^ and memory Th2 cells. The effects of GATA3 knockdown on effector Th2-1^st^ (A), Th2-4^th^ (B) and memory Th2 cells (C) were determined with qRT-PCR. The relative expression (GATA3/Control siRNA) levels are shown. The expression data were rank-ordered from genes with the highest to the lowest relative expression ratios (GATA3/Control siRNA) in Th2-1^st^ cells. The filled squares indicate genes that showed decreases in GATA3 dependency in Th2-memory cells compared with that observed in Th2-1^st^ cells. The open squares indicate genes that showed increases in GATA3 dependency in Th2-memory cells compared with that observed in Th2-1^st^ cells. The lower dashed line indicates 10^-1/2^ and the upper dashed line indicates 10^1/2^.

Finally, we assessed the effects of GATA3 knockdown on *in vivo* generated antigen-specific memory Th2 cells [24]. We analyzed 65 genes shown in Figs. 1–5, and the results in memory Th2 cells (lower panel in Fig. 6) together with those in Th2-1^st^ and Th2-4^th^ cells (upper and middle panels in Fig 6) are summarized. In Th2-1^st^ cells, five of 65 (7.7%) genes showed substantially increased expression, and 16 of 65 (24.6%) genes showed substantially decreased expression by GATA3 knockdown. In Th2-4^th^ cells, one of 65 (1.5%) genes showed substantially increased expression and eight of 65 (12.3%) genes showed substantially decreased expression due to GATA3 knockdown. In memory Th2cells, three of 65 (4.6%) genes showed substantially increased expression and 12 of 65 (18.5%) genes showed substantially decreased expression. The expression levels of Th2 cytokines such as Il-4, Il-5 and Il-13 in memory Th2 cells decreased by GATA3 knockdown. We found that GATA3 dependency was increased by more than two-fold in the Epas1, Cxcr4 and Il-24 genes (red) in memory Th2 cells compared to that observed in Th2-1^st^ cells. We also found that GATA3 dependency was decreased by more than two-fold in the Clcf1, Tube1, Rnf128, Cxcl3, Ccr8 and Il-5 genes (blue). These results indicate that GATA3 regulates the expression of the majority of Th2-specific genes, not only in fully developed Th2 cells but also in *in vivo* generated antigen-specific memory Th2 cells.

## Discussion

We performed a microarray analysis in effector Th2 and fully developed Th2 cells with GATA3 knockdown in order to understand the role of GATA3 in the maintenance of the Th2 cell phenotype. We also developed a new method combining microarray data and ChIP-Seq data to identify transcriptionally and epigenetically activated Th2-specific genes. Consequently, we showed that GATA3 continuously positively or negatively regulates many genes in fully developed Th2 cells. Several interesting features of the transcriptional regulation of Th2 cytokine genes were revealed. Although the importance of GATA3 in the maintenance of the Th2 cell phenotype was confirmed, GATA3 dependency differed for different cytokine genes. For example, the Il-4 expression decreased by 50% following treatment with GATA3 siRNA in all of the Th2 type cells. Previous reports have shown that GATA3 regulates Il-4 expression epigenetically, not transcriptionally [12], [25]. This may explain the relatively weak effects of GATA3 knockdown on Il-4 expression observed in this study.

Il-5 is one of the genes with the highest dependency on GATA3, probably because it is directly transcriptionally regulated by GATA3 [26]. Another feature of Il-5 expression is that its dependency on GATA3 changes in fully developed Th2 cells. This might be explained by the fact that the expression levels of Il-5 tend to increase with the culture cycle number. It is also likely that other transcription factors positively regulate the Il-5 gene expression, because GATA3 dependency was decreased in the Il-5 gene. Il-13, Cxcl3 and Cxcr6 gene expression showed similar behavior to Il-5 expression, and it can be speculated that these four genes are controlled by a similar mechanism (Fig. 5).

We also investigated whether GATA3 is required to maintain the expression of Th2-specific genes other than Th2 cytokine genes. Our new method combining microarray data and ChIP-Seq data enabled us to perform efficient screening for transcriptionally and epigenetically activated Th2-specific genes (Fig. 1A). Downregulation of the gene expression levels by GATA3 siRNA was generally observed in GATA3 target genes in the ChIP-Seq analysis. In a previous report, we demonstrated that GATA3 is sufficient to induce some target genes, which we defined as GATA3 high response genes [22]. In the current study, we assessed all of these GATA3 high response genes (Il-4, Il-5, Il-13, Il-24, Ccnjl, Cyp11a1, Tmtc2, Asb2 and Jdp2 are shown in Fig. 1B) and found that they are greatly influenced by GATA3 knockdown. The expression levels of Il-5, Il-13, Ccnjl, Cyp11a1, Tmtc2 and Asb2 substantially decreased to less than 31.6% (10^-1/2^) in GATA3 knockdown cells (Fig. 1B). In the GATA3 low response group, Ccr8 showed high dependence on GATA3, thus suggesting that GATA3 is necessary, but not sufficient, for Ccr8 expression. In contrast, GATA3 knockdown increased the expression of some Th2-specific genes. Substantial derepression of Tube1 transcription was observed, although Tube1 was highly expressed in Th2 cells. GATA3 may negatively regulates the expression of this gene in Th2 cells.

We identified 55 genes positively regulated by GATA3 in both Th2 and Th2-4^th^ cells. We also identified 58 negatively regulated by GATA3 in both Th2 and Th2-4^th^ cells. Moreover, a total of 365 (55+126+184) genes were classified as being positively regulated by GATA3 in Th2 or Th2-4^th^ cells and a total of 484 (58+260+166) genes were classified as being negatively regulated by GATA3 in Th2 or Th2-4^th^ cells (Fig. 2A). With regard to Th2 cells, 30.4% (55/181) of positively regulated genes and 18.2% (58/318) of negatively regulated genes overlapped with those of Th2-4^th^ cells. Thus, Th2 cells and fully developed Th2 cells share a considerable number of common GATA3-regulated genes. The classification of the 55 common positively regulated genes identified using the method shown in Fig. 2 indicated that 10 genes are directly regulated by GATA3. In the same way, we determined that GATA3 directly regulates seven of the 58 common negatively regulated genes. Preferential expression in Th1 cells and derepression by GATA3 knockdown in Th2 cells were observed in seven extracted Th1 high genes. In contrast, high expression in Th2 cells and decreased gene expression induced by GATA3 siRNA were observed in 10 extracted Th2 high genes (Fig. 2C). These results confirm the validity of our methods.

Cytokines and cytokine receptors are essential effector molecules required for Th cells to exert adequate immune responses. We investigated whether GATA3 regulates cytokine and cytokine receptor genes. In the present study, the well-known Th2 cytokine genes Il5, Il13 and Il24 were detected by a microarray analysis. We also identified many genes that were previously not known to be related to GATA3 in a comparative analysis of Th1 and Th2 cells. The fact that Il6 transcription is regulated by GATA3 is one of the interesting findings in this study. GATA3 knockdown reduced Il6 transcription in Th1, Th2-1^st^ and Th2-4^th^ cells, indicating that Il6 transcription is highly dependent on GATA3. IL-6 plays an active role in inflammation and was recently reported to be a key factor necessary for Th2 cell differentiation [27]. In addition, a recent study reported that STAT3 plays a critical role for in the development and maintenance of human T cell memory [28]. Thus, molecular crosstalk between IL-6/STAT3 and IL-4/STAT6/GATA3 pathway could be an interesting theme to explore.

IL-33 is a member of the IL-1 family, and is expressed in a variety of cells including fibroblasts, epithelial cells, endothelial cells and adipocytes [29]. The administration of IL-33 induces Th2 cytokine production (IL-5 and IL-13) in natural helper cells [30], [31]. Our current data showed that Il33 mRNA is detected in Th2 cells and negatively regulated by GATA3. However, how Il33 transcription is controlled remains unclear, as GATA3 binding was not detected around the Il33 gene locus with ChIP-Seq.

The Lta and Ltb genes encode tumor necrosis factor family proteins [32]. Both are located on Chr17 and are more highly expressed in Th1 cells than Th2 cells. The observation that GATA3 knockdown in Th2 cells causes a de-repression of the expression levels of Lta and Ltb suggests that these genes are typical targets of GATA3-mediated repression. In contrast, the expression levels of Lta and Ltb were not found to be de-repressed by GATA3 knockdown in Th1 cells. We concluded that the GATA3-mediated repression of Lta and Ltb expression is Th2-specific. Furthermore, we found three GATA3 binding peaks around the Lta and Ltb gene loci. These findings indicate that GATA3 directly represses Lta and Ltb expression.

CCL2, also known as monocyte chemotactic protein-1 (MCP-1), recruits monocytes, memory T cells and dendritic cells to sites of tissue injury, infection and inflammation [33]. It is primarily secreted by monocytes, macrophages and dendritic cells. Our data revealed that the Ccl2 transcriptional level in CD4 T cells is generally low, however, it is increased by GATA3 knockdown. This derepression was observed in both Th1 and Th2 cells, indicating that GATA3-mediated repression of Ccl2 transcription is common in CD4 T cell subsets and may prevent excessive immune responses. GATA3 may indirectly repress Ccl2 transcription because there are no GATA3 binding peaks around the Ccl2 gene locus.

We previously reported that both high-level expression of GATA3 and the ability to produce large amount of Th2 cytokine are maintained in memory Th2 cells. [12] In the current report, we confirmed that GATA3 expression is critical for the expression of a certain set of Th2-specific cytokine genes in memory Th2 cells. The effects of GATA3 knockdown on memory Th2 cells were nearly identical to those observed in Th2-1^st^ and Th2-4^th^ cells, although dependency changes were also observed in some genes in memory Th2 cells compared with that observed in Th2-1^st^ cells. GATA3 dependency was found to be decreased in the Clcf1, Tube1, Rnf128, Cxcl3, Ccr8 and Il-5 genes. On the other hand, GATA3 dependency was found to be increased in the Epas1, Cxcr4 and Il-24 genes. However, the fact that only a small number of genes showed change in GATA3 dependency indicates that GATA3-mediated gene regulation mechanisms are similar between Th2-1^st^ and memory Th2 cells.

In summary, our present study revealed that the continuous expression of GATA3 is required for the maintenance of the expression of the majority of Th2-specific genes in fully developed Th2 cells and in vivo generated antigen-specific memory Th2 cells. A list of 32 Th2-specific genes is shown in Table 1. GATA3 binding, expression levels and histone acetylation states for all RefSeq genes are shown in Table S1.

**Table 1:** Overview of 32 Th2-specific inducible genes, highlighting their functions and GATA3 dependency.

RefSeq ID	Genesymbol	h*GATA3*-RV inStat6^−/−^ Th2-1^st^ (*)^22^	*Gata3* KD inTh2-1^st^ (**)	*Gata3* KD inTh2-4^th^ (**)	*Gata3* KD inmemory Th2 (**)	GATA3 dependency inmemory Th2	GO term
GATA3 target genes							
NM_023049	*Asb2*	92.92	21.44	34.30	28.27		ank repeat, ubl conjugation pathway
NM_001045530	*Ccnjl*	42.66	19.81	23.19	unevaluable		cyclin
NM_007720	*Ccr8*	19.44	7.22	11.93	45.30	decrease	chemokine receptor activity
NM_013498	*Crem*	27.90	72.53	70.15	77.20		transcription factor activity
NM_019779	*Cyp11a1*	122.42	18.08	21.90	28.18		cholesterol metabolism, steroid metabolism
NM_010137	*Epas1*	−0.06	74.05	75.86	26.93	increase	transcription factor activity
NM_008091	*Gata3*	12.35	18.87	17.37	23.05		transcription factor activity
NM_010370	*Gzma*	-5.74	51.85	40.70	51.02		cytolysis, Serine protease
NM_008355	*Il13*	201.35	11.90	25.56	21.29		cytokine activity
NM_053095	*Il24*	41.26	61.95	57.28	3.92	increase	cytokine activity
NM_021283	*Il4*	50.77	38.97	49.07	26.87		cytokine activity
NM_010558	*Il5*	75.90	1.40	4.41	3.63	decrease	cytokine activity
NM_016780	*Itgb3*	26.34	86.97	98.28	43.46		cell adhesion
NM_030887	*Jdp2*	78.41	52.77	30.03	87.20		transcription factor activity
NM_017373	*Nfil3*	−2.32	26.62	49.06	48.89		transcription factor activity
NM_177368	*Tmtc2*	55.56	20.90	35.14	19.10		transmembrane
NM_009401	*Tnfrsf8*	−16.18	91.68	119.09	95.09		receptor
GATA3 non-target genes							
NM_176933	*Dusp4*	-8.20	133.15	97.16	107.88		protein phosphatase
NM_007899	*Ecm1*	2.16	59.75	124.92	49.03		extracellular matrix
NM_010169	*F2r*	29.09	49.36	40.11	41.84		thrombin receptor activity, blood coagulation
NM_025768	*Grtp1*	9.13	unevaluable	unevaluable	unevaluable		GTPase activation
NM_010555	*Il1r2*	−21.22	43.46	60.71	84.63		cytokine receptor activity
NM_013871	*Mapk12*	7.27	43.84	69.52	40.36		MAP kinase activity
NM_010959	*Oit3*	unevaluable	unevaluable	unevaluable	unevaluable		calcium ion binding
NM_001002927	*Penk*	10.21	56.13	51.23	69.82		opioid peptide activity
NM_019676	*Plcd1*	−14.71	98.15	73.64	95.92		phosphoinositide phospholipase C activity
NM_008967	*Ptgir*	110.02	29.08	47.17	32.11		g-protein coupled receptor
NM_023270	*Rnf128*	20.85	24.75	39.68	70.82	decrease	transmembrane, ubl conjugation pathway
NM_011309	*S100a1*	18.57	74.10	84.12	71.13		calcium ion binding
NM_011897	*Spry2*	unevaluable	145.42	72.15	66.60		developmental protein, membrane
NM_181071	*Tanc2*	26.89	35.85	45.44	53.09		ank repeat
NM_028006	*Tube1*	−21.52	324.17	199.93	88.45	decrease	cytoplasm, cytoskeleton

(*) GATA3 dependency was evaluated by the recovery score (RS) [22].

(**) GATA3 dependency was evaluated by gene expression ratio (Gata3/Control siRNA).

“Unevaluable” means that mRNA level was too low to detect by qPCR.

Unifying 24 genes identified in the present study and previously reported 26 genes [22], 32 genes were defined as Th2-specific inducible genes. Four biological readouts in these 32 genes are shown; (1) changes in gene expression by enforced expression of hGATA3 in STAT6-deficient Th2-1^st^ cells (3^rd^ column), (2) changes in gene expression by GATA3 knockdown in Th2-1^st^ cells (4^th^ column), (3) those in Th2-4^th^ cells (5^th^ column), and (4) those in memory Th2 cells (6^th^ column). In the 3^rd^ column, GATA3 dependency was determined by the recovery score (RS), which is defined as the linear equation below [22]:

RS (%)  = 100×(C−B)/(A−B).

where *A* indicates the signal intensity of Th2, *B* indicates the signal intensity of STAT6-deficient Th2, and *C* indicates the signal intensity of STAT6-deficient Th2 with overexpression of hGATA3. In the 4^th^, 5^th^, and 6^th^ column, GATA3 dependency was determined by gene expression ratio (*Gata3*/Control siRNA). Gene ontology (GO) terms identified by DAVID analysis tool are shown in the 7^th^ column.

## Supporting Information

Figure S1 **The effects of GATA3 knockdown on the expression of Th2-specific genes.** The gene expression levels of GATA3, 16 Th2-specific GATA3 target genes and 15 Th2-specific GATA3 non-target genes in Th1 cells and Th2 cells were determined with qRT-PCR. The relative intensity (relative expression/Hprt; highest signal intensity  = 10) (mean of two samples) levels are shown.
(TIF)

Figure S2 **The effects of GATA3 knockdown on the expression of GATA3 protein. (A),** The protein expression of GATA3 was determined by immunoblotting with specific mAbs. The protein expression of Tubulin was shown as an internal control. **(B),** The levels of GATA3 protein expression were determined by intracellular staining.
(TIF)

Figure S3 **The effects of GATA3 knockdown on the expression of potential GATA3-regulated genes in Th2 cells.** The gene expression levels of GATA3, six genes negatively regulated by GATA3 (Th1 high genes) and nine genes positively regulated by GATA3 (Th2 high genes) in Th1 cells and Th2 cells were determined with qRT-PCR. The relative intensity (relative expression/Hprt; highest signal intensity  = 10) (mean of two samples) levels are shown.
(TIF)

Figure S4 **The effects of GATA3 knockdown on the expression of cytokine and cytokine receptor genes.** The gene expression levels of GATA3, 12 cytokine genes positively regulated by GATA3, 12 cytokine genes negatively regulated by GATA3, seven cytokine receptor genes positively regulated by GATA3 and four cytokine receptor genes negatively regulated by GATA3 in Th1 cells and Th2 cells were determined with qRT-PCR. The relative intensity (relative expression/Hprt; highest signal intensity  = 10) (mean of two samples) levels are shown.
(TIF)

Figure S5 **A comparison of GATA3 dependency among Th2-1^st^ and Th2-4^th^ cells in a 2-D plot.** The gene expression profiles studied with the microarray analysis were analyzed in a 2-D plot. The x-axis shows the relative expression (GATA3/Control siRNA) levels in Th2-1st cells. The y-axis shows the relative expression (GATA3/Control siRNA) levels in the Th2-4^th^ cells.
(TIF)

Figure S6 **Analysis of GATA3 binding at Th2-specific genes.** A ChIP assay was performed using anti-GATA3 antibody and control anybody in Th2-1^st^, Th2-2^nd^, Th2-3^rd^ and Th2-4^th^ cells. The levels of binding at the indicated GATA3-bound Th2-specific genes were assessed by a qPCR analysis and shown in percent input (Specific Ab ChIP/input DNA; mean of two samples). The binding region in the Gzma gene was unable to be included because of difficulties encountered during the design of qPCR primers specific for this region. Detail information about GATA3-bound regions is available in a previous report [22].
(TIF)

Table S1 **GATA3 binding, expression levels and histone acetylation states for all RefSeq genes.**
(XLS)

Materials and Methods S1 (DOCX)
