# Supplementary figures and images for: Genome-Wide Gene Expression Profiling Revealed a Critical Role for GATA3 in the Maintenance of the Th2 Cell Identity
Source: PLoS One. 2013 Jun 18;8(6):e66468. doi: 10.1371/journal.pone.0066468 (PMC3688927; doi:10.1371/journal.pone.0066468)

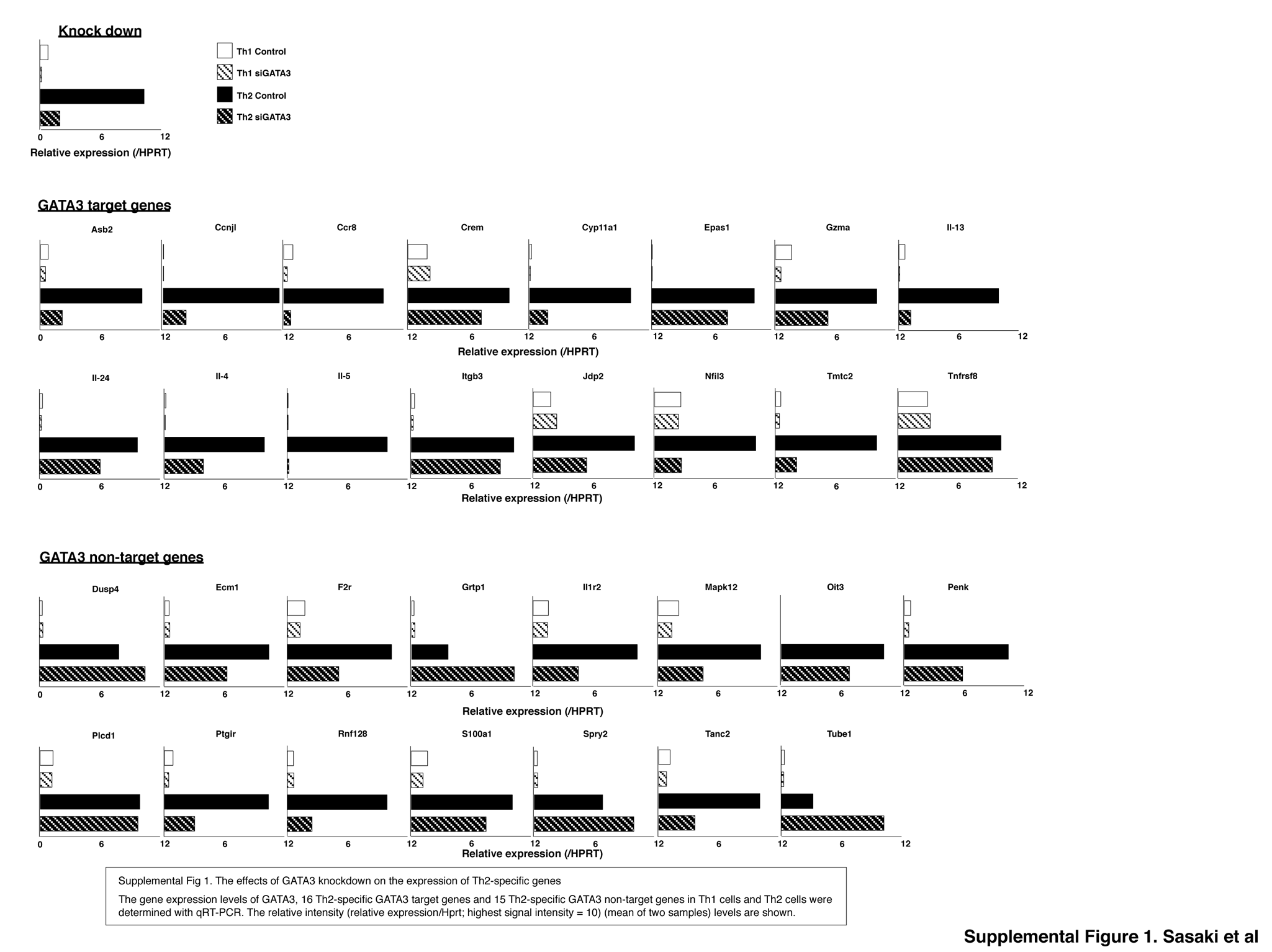

Supplement: Figure S1 — The effects of GATA3 knockdown on the expression of Th2-specific genes. The gene expression levels of GATA3, 16 Th2-specific GATA3 target genes and 15 Th2-specific GATA3 non-target genes in Th1 cells and Th2 cells were determined with qRT-PCR. The relative intensity (relative expression/Hprt; highest signal intensity = 10) (mean of two samples) levels are shown. (TIF) [file pone.0066468.s001.tif]

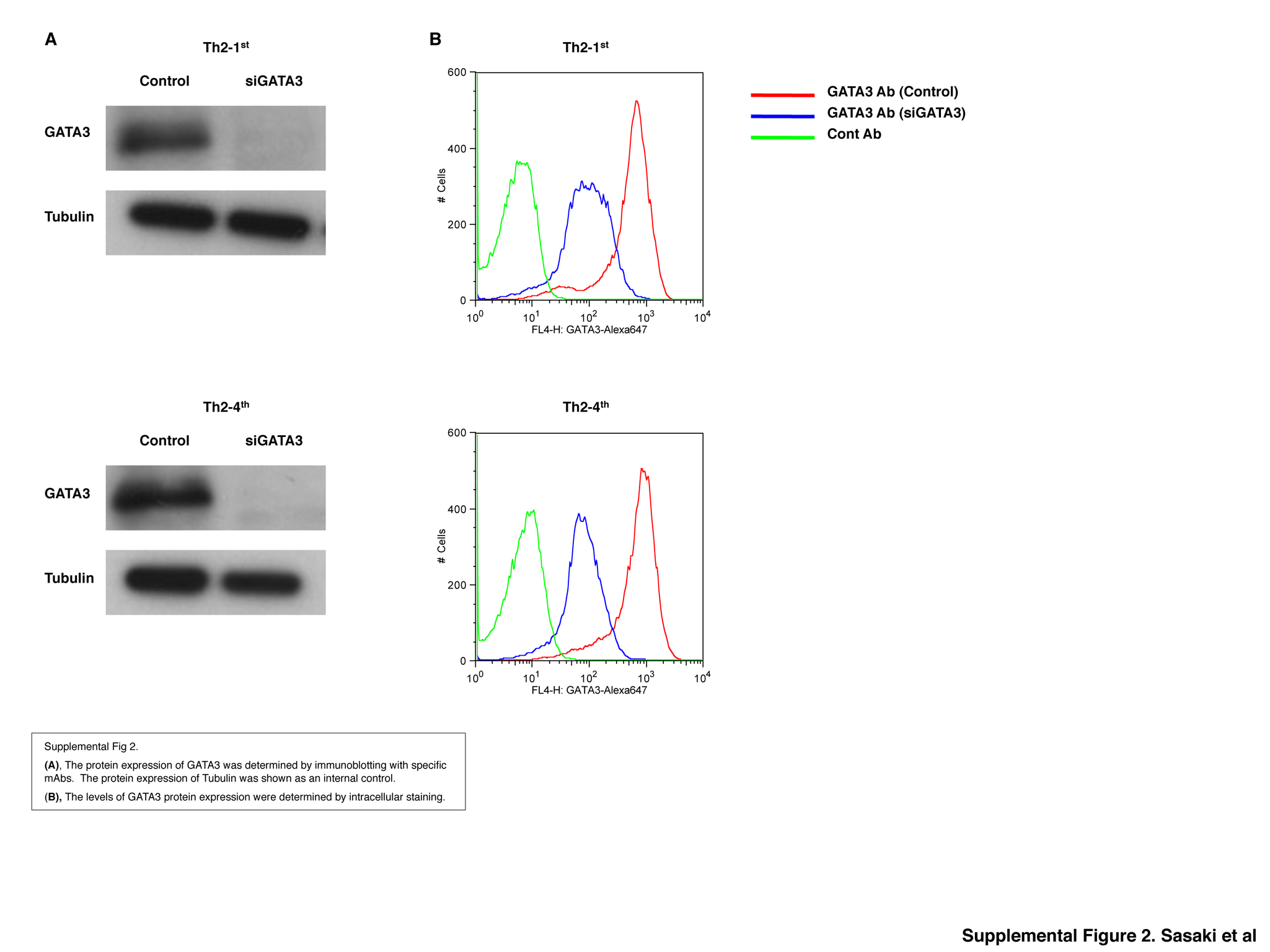

Supplement: Figure S2 — The effects of GATA3 knockdown on the expression of GATA3 protein. (A), The protein expression of GATA3 was determined by immunoblotting with specific mAbs. The protein expression of Tubulin was shown as an internal control. (B), The levels of GATA3 protein expression were determined by intracellular staining. (TIF) [file pone.0066468.s002.tif]

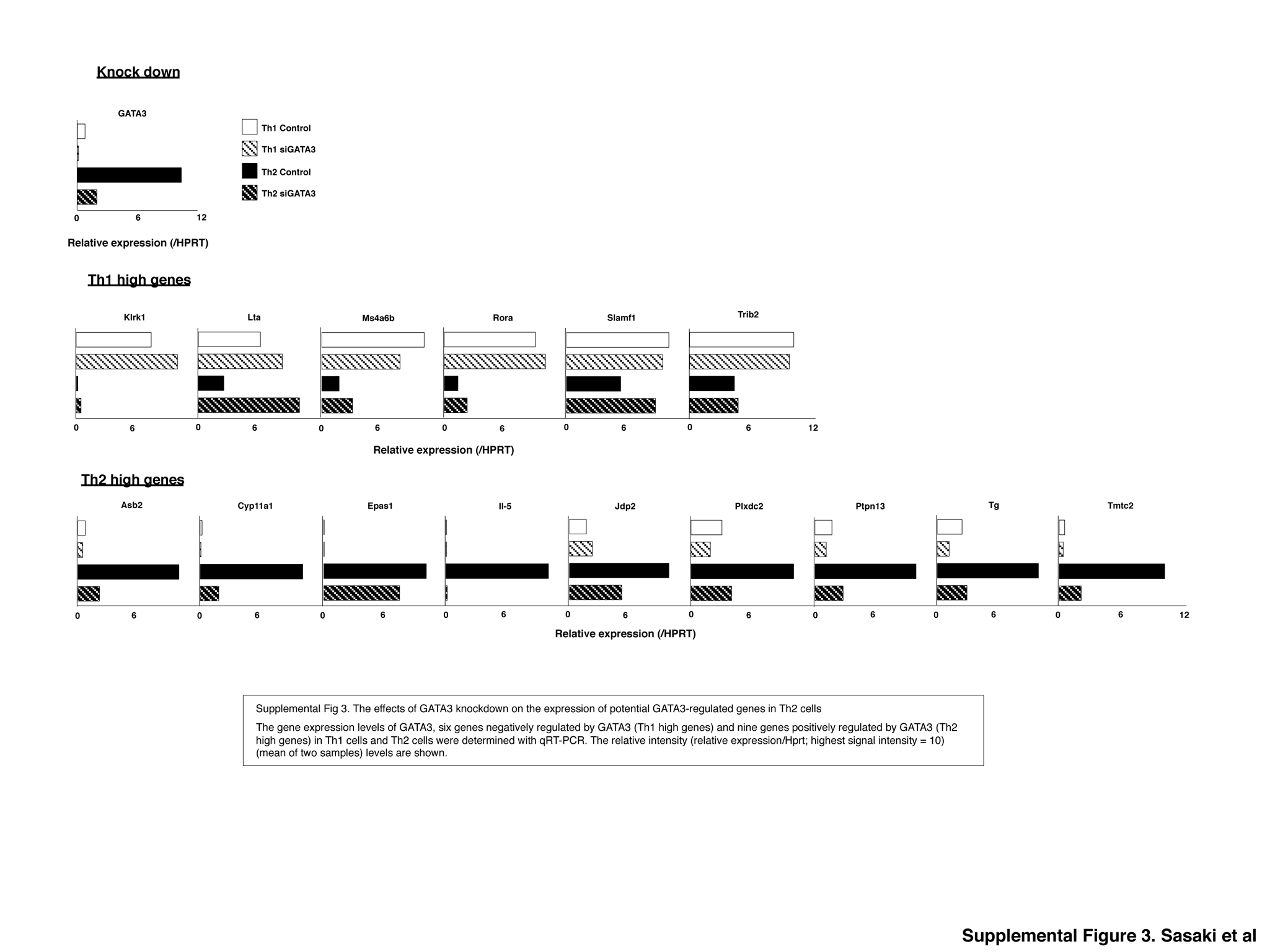

Supplement: Figure S3 — The effects of GATA3 knockdown on the expression of potential GATA3-regulated genes in Th2 cells. The gene expression levels of GATA3, six genes negatively regulated by GATA3 (Th1 high genes) and nine genes positively regulated by GATA3 (Th2 high genes) in Th1 cells and Th2 cells were determined with qRT-PCR. The relative intensity (relative expression/Hprt; highest signal intensity = 10) (mean of two samples) levels are shown. (TIF) [file pone.0066468.s003.tif]

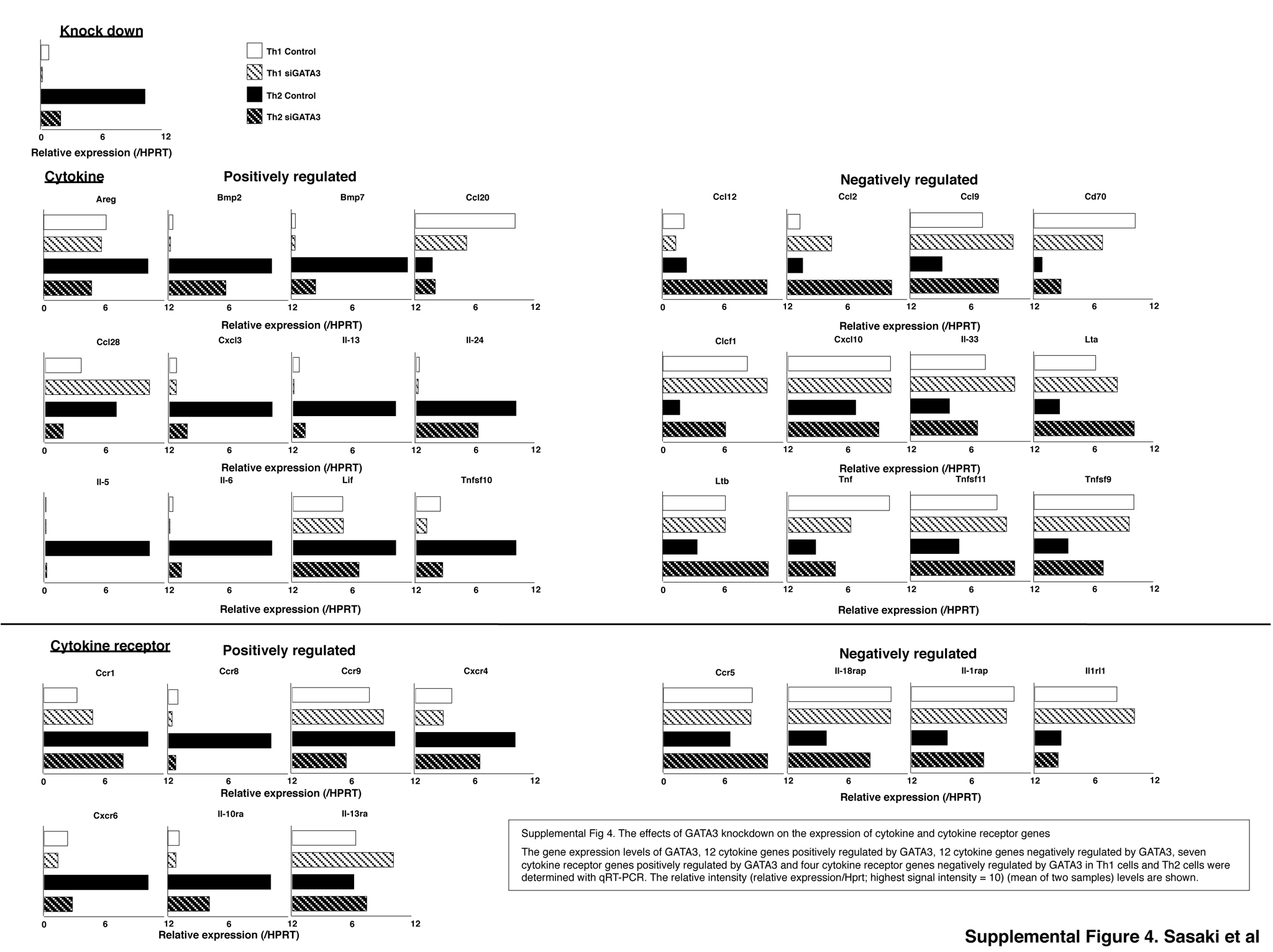

Supplement: Figure S4 — The effects of GATA3 knockdown on the expression of cytokine and cytokine receptor genes. The gene expression levels of GATA3, 12 cytokine genes positively regulated by GATA3, 12 cytokine genes negatively regulated by GATA3, seven cytokine receptor genes positively regulated by GATA3 and four cytokine receptor genes negatively regulated by GATA3 in Th1 cells and Th2 cells were determined with qRT-PCR. The relative intensity (relative expression/Hprt; highest signal intensity = 10) (mean of two samples) levels are shown. (TIF) [file pone.0066468.s004.tif]

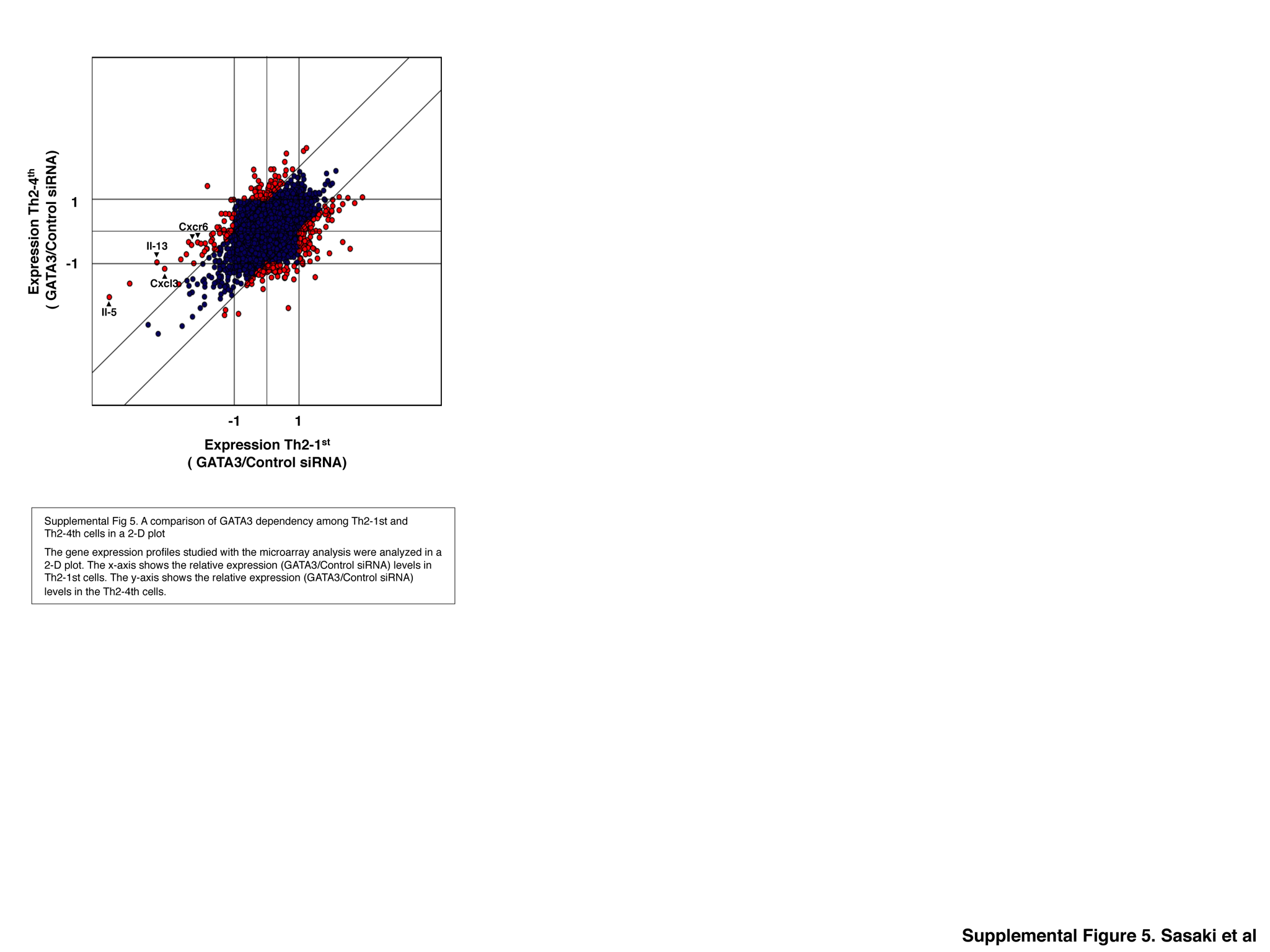

Supplement: Figure S5 — A comparison of GATA3 dependency among Th2-1st and Th2-4th cells in a 2-D plot. The gene expression profiles studied with the microarray analysis were analyzed in a 2-D plot. The x-axis shows the relative expression (GATA3/Control siRNA) levels in Th2-1st cells. The y-axis shows the relative expression (GATA3/Control siRNA) levels in the Th2-4th cells. (TIF) [file pone.0066468.s005.tif]

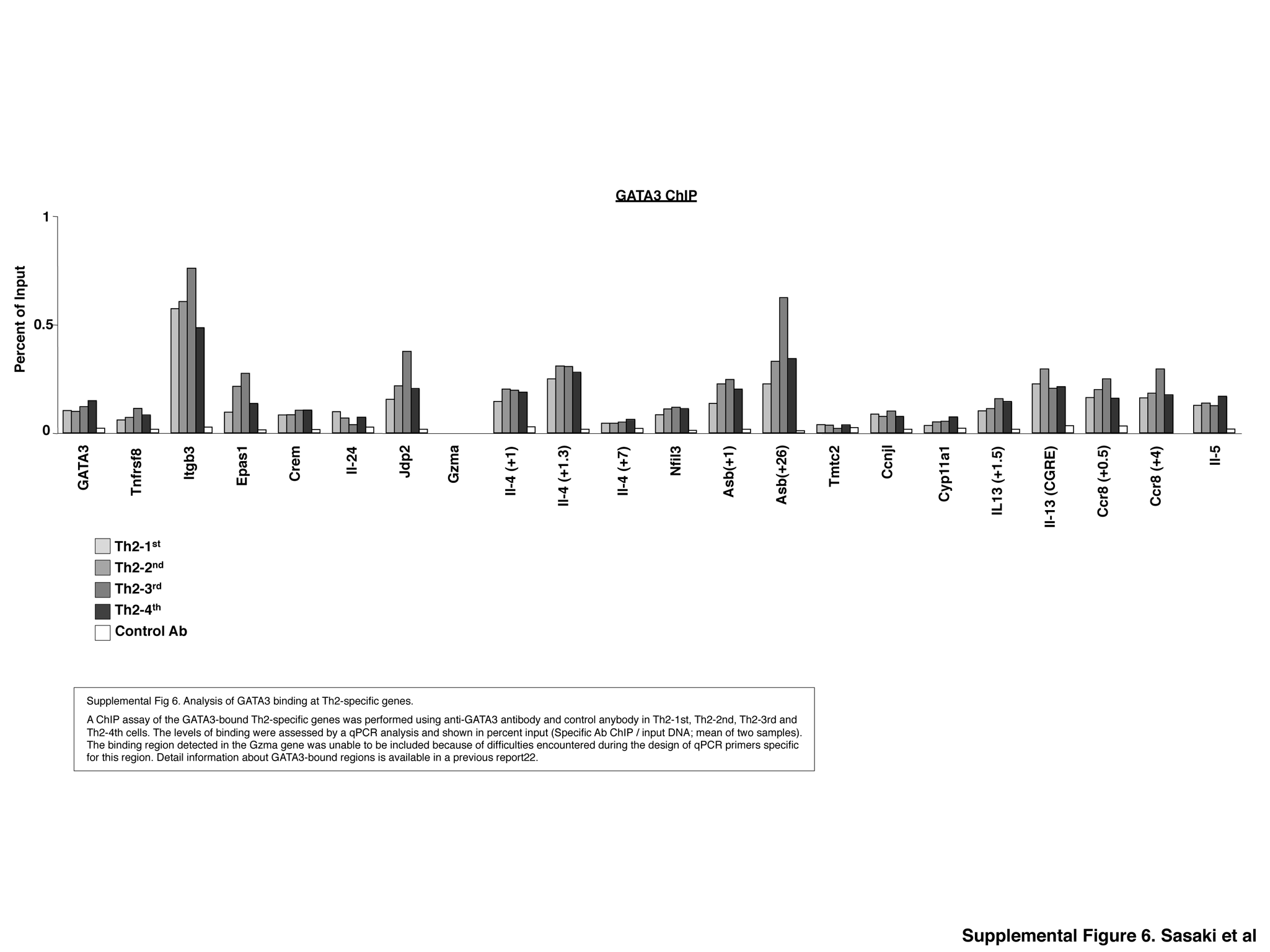

Supplement: Figure S6 — Analysis of GATA3 binding at Th2-specific genes. A ChIP assay was performed using anti-GATA3 antibody and control anybody in Th2-1st, Th2-2nd, Th2-3rd and Th2-4th cells. The levels of binding at the indicated GATA3-bound Th2-specific genes were assessed by a qPCR analysis and shown in percent input (Specific Ab ChIP/input DNA; mean of two samples). The binding region in the Gzma gene was unable to be included because of difficulties encountered during the design of qPCR primers specific for this region. Detail information about GATA3-bound regions is available in a previous report [22]. (TIF) [file pone.0066468.s006.tif]
