# Supplementary material for: Genome-Wide Gene Expression Profiling Revealed a Critical Role for GATA3 in the Maintenance of the Th2 Cell Identity
Source: PLoS One. 2013 Jun 18;8(6):e66468. doi: 10.1371/journal.pone.0066468 (PMC3688927; doi:10.1371/journal.pone.0066468)
Supplement: Materials and Methods S1 — (DOCX) [file pone.0066468.s008.docx]

*Immunoblot Analysis*

Cytoplasmic extracts and nuclear extracts were prepared using NE-PER Nuclear and Cytoplasmic Extraction Reagent (Pierce Chemical Co., CAT#78833). The antibodies used for the immunoblot analysis were anti-GATA3 (Santa cruz: sc-268),　anti-Tubulin alpha (NeoMarkers: DM1A).

*Immunofluorescent staining and flow cytometric analysis*

Intracellular staining was also performed as described previously[17]. Anti-GATA3-alexa647 (BD Pharmingen 560068) antibody was used for cytoplasmic staining. The flow cytometric analysis was performed on a FACSCalibur instrument (BD Biosciences, San Jose, CA) and the results were analyzed with the FlowJo software program (BD Biosciences).
